# Refractive Errors and Educational Outcomes in Children: A Systematic Review

**DOI:** 10.7759/cureus.107531

**Published:** 2026-04-22

**Authors:** Amna Dafaalla Hasab Elrasoul Elbasheer, Batoul Mahmoud MohammedAhmed Suliman, Mohamed kbashe Mohamed Bahr, Elhareth Haydar Awad Abdelhalim, Yaser Elhams, Abla Almalik, Asim Ahmed

**Affiliations:** 1 Ophthalmology, Al Azhar Hospital, Riyadh, SAU; 2 Ophthalmology, Response Plus Medical Complex, Dammam, SAU; 3 Ophthalmology, Faculty of Medicine, Karary University, Khartoum, SDN; 4 Faculty of Medicine, Karary University, Khartoum, SDN; 5 Ophthalmology, Umm Al-Qura University, Makkah, SAU; 6 Ophthalmology, Alshaea Hospital, Ar Rass, SAU; 7 Medicne and Surgery, University of Gezira, Wad Madani, SDN

**Keywords:** astigmatism, hyperopia, myopia, refractive errors, spectacles

## Abstract

Refractive errors such as hyperopia, myopia, and astigmatism are common in childhood. They may interfere with learning when uncorrected, particularly when near blur and increased accommodative demand reduce reading efficiency and sustained attention. We conducted a systematic review of observational and interventional studies evaluating associations between refractive errors and cognitive or academic outcomes in children and adolescents. PubMed, Embase, and Scopus were searched from inception to January 2025, and reference lists of eligible articles were hand-searched. Twenty-five studies were included: 16 cross-sectional or other nonrandomized analytical studies, 4 cohort studies, and 5 randomized studies. The included studies spanned preschool and school-aged populations across diverse geographic settings and assessed outcomes including early literacy, attention, visual-motor integration, reading speed, grades, and standardized academic performance. Across preschool studies, uncorrected hyperopia showed a consistent association with poorer early literacy and attention-related outcomes. In school-based studies, observational evidence generally linked uncorrected refractive error or reduced habitual visual acuity to lower academic attainment. Interventional and randomized evidence suggests that spectacle provision can produce modest improvements in reading efficiency and mathematics performance in some settings, including gains of approximately 0.1 standard deviations in some cluster-randomized trials. However, effects varied across programs and were often sensitive to adherence, timing of outcome assessment, implementation intensity, and outcome measurement. Heterogeneity in exposure definitions, outcome measures, and reporting limited quantitative pooling and supports cautious interpretation. Risk of bias was mainly related to confounding in observational studies, limited masking and adherence in randomized studies, and residual uncertainty across heterogeneous evidence. Overall, the evidence indicates that timely detection and correction of refractive errors may support children’s learning, although certainty is limited by heterogeneity, risk of bias, and inconsistent outcome reporting. Future studies should prioritize standardized educational outcomes, rigorous control of confounding, and sustained strategies to optimize spectacle wear.

## Introduction and background

Refractive errors, including hyperopia, myopia, and astigmatism, are among the most common causes of avoidable vision impairment worldwide and remain highly prevalent in childhood, a developmental period during which visual input is foundational for learning. The World Health Organization has emphasized that uncorrected refractive error is a major, addressable contributor to vision impairment and that strengthening school- and community-based eye care is essential to reduce lifelong educational and social disadvantage [1]. Global estimates from 2000 to 2020 indicate that a substantial proportion of visual impairment is attributable to uncorrected refractive errors [2], with broader societal consequences, including productivity losses when vision problems remain untreated [3]. The epidemiology of pediatric refractive error is also changing, particularly for myopia, with recent global projections indicating a continued rise in prevalence among children and adolescents in the coming decades [4]. Hyperopia is likewise common in early childhood, and meta-analytic estimates indicate meaningful prevalence across pediatric age groups, with variability by geography and study methods [5]. Population-based studies among schoolchildren show that clinically significant refractive error and reduced presenting visual acuity are common, reinforcing the need to evaluate functional consequences beyond ophthalmic endpoints [6]. This burden is especially relevant in school-based, resource-constrained settings, where delayed detection, incomplete referral, limited access to spectacles, and inconsistent wear may allow correctable visual problems to persist during critical stages of learning.

The pathway from refractive error to learning outcomes is biologically plausible. In young children, moderate hyperopia can degrade near-retinal image quality and increase accommodative demand during sustained near tasks such as letter identification, early reading, and writing. These factors may lead to visual discomfort, reduced attention, and poorer task efficiency. Cohort-nested evidence has linked reduced visual acuity at the start of schooling with weaker literacy development, suggesting that even modest reductions in functional vision may affect education during critical learning periods [7]. Experimental and clinical studies also indicate that hyperopic children may exhibit accommodative lag and less stable near focus than emmetropic peers, supporting a mechanism by which persistent near blur could interfere with early literacy acquisition [8]. However, this pathway should be interpreted alongside developmental, socioeconomic, and school-level factors, all of which can influence access to visual correction and educational performance.

Beyond biological mechanisms, the evidence linking refractive error to educational outcomes is expanding but remains heterogeneous across settings, refractive types, and outcome measures. A recent systematic review and meta-analysis concluded that hyperopia is associated with poorer academic performance and reading-related outcomes; however, effect sizes vary depending on hyperopia thresholds, correction status, and assessment tools [9]. This review extends earlier work by synthesizing both observational and interventional evidence on spectacle correction and service delivery. These two evidence streams address different but complementary questions. Observational studies help determine whether uncorrected refractive error is associated with poorer learning outcomes in real-world populations, whereas interventional studies assess whether detection and correction can modify those outcomes. The translation of vision care into educational benefits also depends on screening quality and downstream care pathways. Recent evidence on school-based eye health interventions in low- and middle-income countries emphasizes that referral, follow-up, spectacle availability, adherence, procurement, and sustainability are critical determinants of impact [10]. Even when spectacles are provided, adherence can be suboptimal, and pooled evidence indicates that spectacle compliance is a key limitation that may reduce real-world effectiveness [11]. Interventional, economic, and public health evaluations nevertheless suggest that reducing access barriers through spectacle provision can improve academic performance in some contexts [12]. Interpretation is further complicated by confounding and bidirectionality, particularly in myopia, where educational exposure may contribute to refractive development, creating potential reverse causality when academic achievement and refractive status are examined cross-sectionally [13]. Regional data from the Eastern Mediterranean indicate a meaningful burden of childhood visual impairment, highlighting the relevance of refractive error as a child health and education issue in the region [14]. This relevance in low- and middle-income countries is important, as educational effects may depend not only on the presence of refractive error, but also on whether children are screened, referred, provided with affordable spectacles, and supported to wear them consistently.

A focused synthesis is needed to clarify whether uncorrected refractive errors are associated with deficits in cognitive, literacy, or academic outcomes in children and adolescents, and whether spectacle correction improves these outcomes in real-world settings. This focus is important because previous research has often examined epidemiologic associations and intervention effects separately, whereas school health decision-making requires both forms of evidence to understand burden, plausibility, and practical benefit. The primary objective of this systematic review is to evaluate whether uncorrected refractive errors are associated with cognitive, literacy, or academic performance outcomes in children and adolescents. The secondary objective is to assess the impact of spectacle correction and related service-delivery factors on these outcomes, with implications for clinical practice, school screening strategies, and child health policy.

## Review

Methods

This systematic review was conducted in accordance with the Preferred Reporting Items for Systematic Reviews and Meta-Analyses (PRISMA) guidelines [15]. A protocol was prepared prior to the literature search and is available from the authors upon request.

We searched PubMed, Embase, and Scopus from database inception through January 31, 2025 (the date of the final search). No language restrictions were applied at the search stage; however, studies were included only if sufficient information was available for eligibility assessment and data extraction. The review included peer-reviewed, published studies, while unpublished studies, dissertations, theses, grey literature, and conference abstracts lacking sufficient data were excluded.

The search strategy combined controlled vocabulary terms (e.g., Medical Subject Headings and Emtree) and free-text keywords related to refractive error, vision screening or correction, and cognitive or educational outcomes in children. The full database-specific search strategies for PubMed, Embase, and Scopus are provided in Supplementary Material 1.

We also hand-searched the reference lists of included studies and relevant reviews to identify additional eligible reports. Two reviewers independently screened titles and abstracts, assessed full texts against eligibility criteria, and extracted data. Discrepancies were resolved by consensus.

Eligibility Criteria

We included studies involving children or adolescents (0-18 years) without known neurological disorders. Eligible exposures were uncorrected refractive errors (hyperopia, myopia, astigmatism, or anisometropia) or interventions providing spectacles to correct these errors. Comparators included emmetropic peers, corrected refractive error, or baseline assessments prior to intervention. The primary outcomes were cognitive, literacy, or academic performance measures, including early literacy scores, reading-related performance, grade-point averages, and standardized educational test scores. Secondary outcomes included attention, visual-motor integration, reading speed or efficiency, spectacle wear, adherence, and service-delivery indicators when reported alongside cognitive or educational outcomes. We included cross-sectional surveys, cohort studies, quasi-experimental studies, and randomized controlled trials. For interventional studies, eligibility required at least one post-intervention outcome assessment following spectacle correction or program implementation. Immediate or short-term post-correction assessments were accepted for functional reading or near-task outcomes, whereas academic outcomes required assessment after the intervention had been implemented in the relevant educational setting. Studies involving adults, syndromic conditions, or exclusively visual outcomes were excluded.

The population, exposure/intervention, comparator, and outcome (PICO) framework was applied in this review. Table 1 outlines the eligibility criteria that guided the study selection.

**Table 1:** PICO framework for inclusion criteria The PICO framework summarizes the population characteristics, exposures or interventions, comparator groups, and outcome domains considered for inclusion in this review.

Component	Definition	Examples
Population	Children and adolescents aged 0-18 years without syndromic or neurological disorders	Preschoolers, primary‑school pupils, adolescents participating in school‑based vision programs
Exposure/intervention	Uncorrected refractive errors (hyperopia, myopia, astigmatism, or anisometropia) and interventions to correct these errors (spectacle provision, vision screening)	Moderate hyperopia ≥+3 D; uncorrected myopia; school vision screening with free glasses
Comparator	Emmetropic peers, corrected refractive error, alternative correction approaches, or baseline pre‑intervention assessments	Emmetropic children in cross‑sectional studies; near‑vision correction vs partial/no correction; control schools in cluster trials
Outcomes	Cognitive and educational measures	Early literacy tests, reading speed, attention or visual-motor integration scores, grade‑point averages, standardized mathematics and language tests.

From each included study, we extracted information on study design, setting, sample size, age, type and severity of refractive error, comparator group, outcomes measured, effect estimates, and conclusions. Screening and data extraction records were managed using a standardized Microsoft Excel spreadsheet (Microsoft Corp., Redmond, WA, USA). Before full screening, reviewers calibrated the eligibility criteria on a sample of records to ensure consistent interpretation of the inclusion and exclusion criteria. Two reviewers independently screened titles and abstracts, assessed full texts against eligibility criteria, and extracted data. Discrepancies were resolved by consensus. Formal inter-rater agreement using kappa statistics was not calculated. Because outcome measures and study designs varied widely, a narrative synthesis was undertaken and reported using principles from the Synthesis Without Meta-analysis (SWiM) guideline [16]. Key characteristics were summarized in tables, and results were organized by outcome domain. The synthesis was structured by study design and outcome category, including early literacy and attention, reading speed and near-task performance, academic attainment, and spectacle correction or service-delivery outcomes. Where available, quantitative effect estimates, adjusted associations, and direction of effect were extracted and summarized in the results tables and narrative text. A meta-analysis was not performed due to heterogeneity in measures and reporting, including variation in refractive error definitions, intervention models, educational outcomes, timing of assessment, and reported effect estimates.

Risk-of-Bias Assessment

We assessed the methodological quality of each included study using design-appropriate tools. For randomized controlled trials, we applied the Cochrane Risk-of-Bias tool, version 2 (RoB 2), in accordance with the Cochrane guidance, to evaluate randomization processes, deviations from intended interventions, missing outcome data, measurement of outcomes, and selective reporting. For nonrandomized interventional studies, we used the Risk of Bias in Non-randomized Studies of Interventions (ROBINS-I) tool according to the official ROBINS-I guidance. Observational cohort studies were appraised using the Newcastle-Ottawa Scale, which assesses participant selection, comparability, and outcome ascertainment, whereas cross-sectional and prevalence studies were appraised using the Joanna Briggs Institute critical appraisal tools. Two reviewers independently performed the risk-of-bias assessments, and disagreements were resolved through discussion. Overall judgments (low, moderate, or high risk of bias) informed the narrative synthesis but did not lead to exclusion of studies. Findings from randomized trials and lower-risk observational studies were given greater interpretive weight, whereas findings from cross-sectional and high-risk nonrandomized studies were interpreted more cautiously. Formal GRADE assessment was considered; however, overall certainty was summarized narratively because the included evidence was methodologically heterogeneous, outcomes were not pooled, and no meta-analysis was performed.

Study Selection

**Figure 1:**
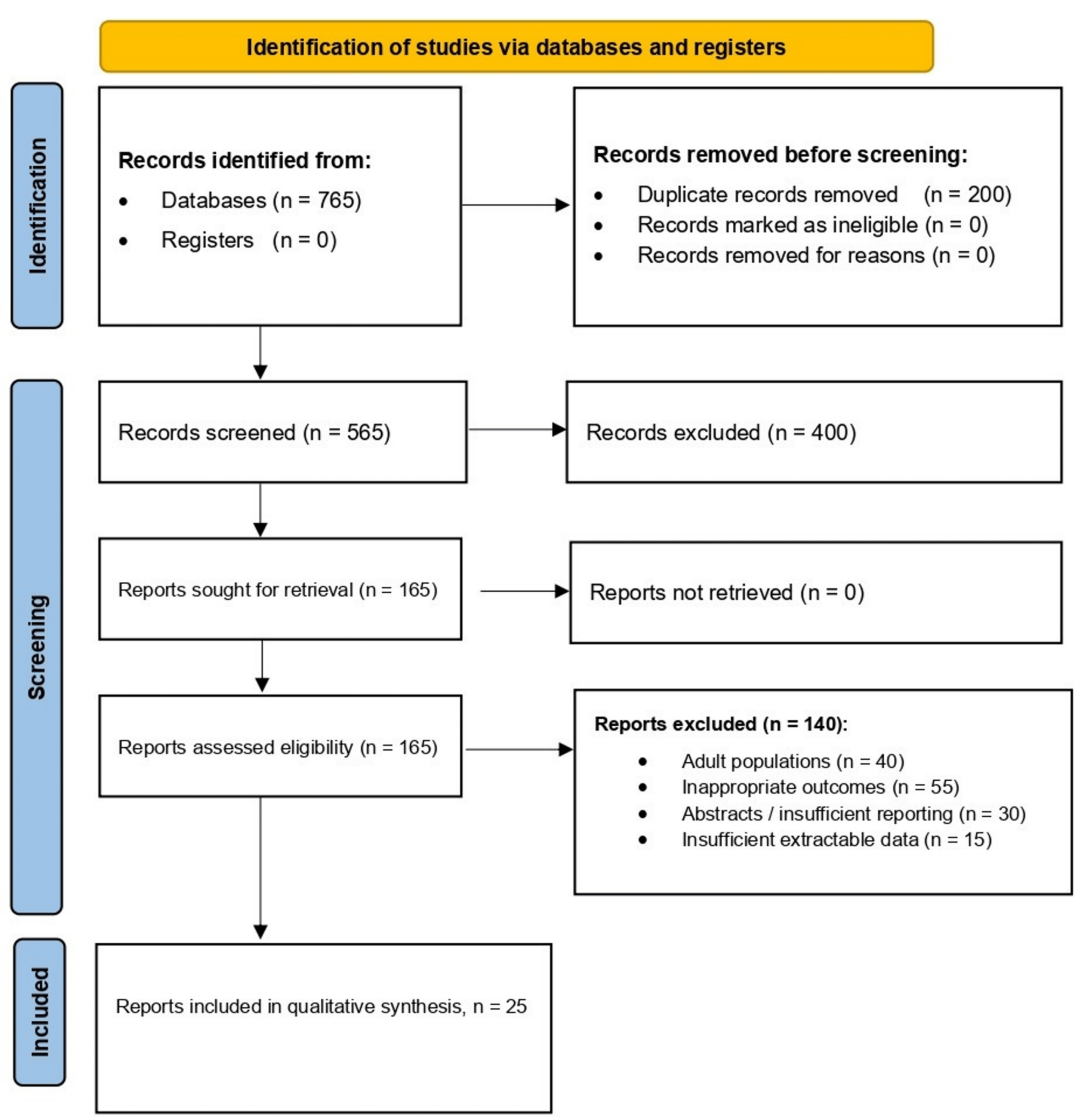
PRISMA flow diagram of study identification, screening, eligibility, and inclusion Records were identified through database searching; duplicates were removed before screening. Titles and abstracts were screened, and full texts of potentially eligible reports were assessed against predefined inclusion and exclusion criteria. Studies meeting eligibility criteria were included in the qualitative synthesis. PRISMA: Preferred Reporting Items for Systematic Reviews and Meta-Analyses.

The electronic search identified 765 records. After removing 200 duplicates, 565 titles and abstracts were screened, and 400 were excluded because they did not meet the eligibility criteria. One hundred sixty-five full-text articles were assessed for eligibility, and 25 studies met the inclusion criteria. Full-text exclusions (n = 140) were primarily due to adult populations (n = 40), inappropriate outcomes (n = 55), conference abstracts or insufficient reporting (n = 30), and insufficient extractable data (n = 15). The completed PRISMA 2020 checklist is provided in Supplementary material 2. Figure 1 presents the PRISMA flow diagram.

Study Characteristics

The 25 included studies [17-41] were classified into three broad design categories: cross-sectional or other nonrandomized analytical studies [17-19,21-23,25-29,31,36,38-40], cohort studies [20,24,32,41], and randomized studies, including cluster randomized trials, a randomized crossover trial, and a randomized field experiment [30,33-35,37]. Collectively, studies were conducted across Asia (including China, Vietnam, Saudi Arabia, Singapore, and Malaysia), Europe (including the United Kingdom and the Netherlands), North America (including the United States), and Africa (including Ethiopia and Nigeria). Definitions of hyperopia and refractive error varied across studies, including moderate hyperopia thresholds and broader definitions of any refractive error. Outcomes included preschool literacy and attention measures, reading speed, standardized test scores, and grade point averages. To improve readability, Table 2 was simplified to highlight key characteristics of each study, including study design, sample size, population or setting, outcome domain, and main findings. Study characteristics and key findings are summarized in Table 2.

**Table 2:** Summary of included studies

Study	Design	Sample and setting	Outcome focus	Main findings
Kulp et al. (2016) [17]	Cross-sectional	492 preschool children	Early literacy and visual function	Uncorrected hyperopia was associated with poorer near-vision function and lower early literacy scores, especially in children with reduced near-visual acuity or stereoacuity
Ntodie et al. (2021) [18]	Within-subject interventional study	63 school-aged children with low-to-moderate hyperopia	Accommodative function and reading speed	Full hyperopic correction improved accommodative accuracy during sustained near work and increased reading speed
Shankar et al. (2007) [19]	Pilot cross-sectional study	31 kindergarten children	Emergent literacy	Hyperopic children had poorer letter and word recognition and receptive vocabulary than non-hyperopic peers
Williams et al. (2005) [20]	Cohort study	UK primary school cohort	Educational attainment	Hyperopia was associated with lower standardized educational attainment
Rosner and Rosner (1997) [21]	Cross-sectional study	782 schoolchildren	Academic achievement	Children with moderate hyperopia had lower achievement scores than emmetropic children
Narayanasamy et al. (2015) [22]	Experimental simulation study	15 children in a laboratory setting	Reading-related performance	Simulated hyperopia reduced performance during reading-related near tasks
Roch-Levecq et al. (2008) [23]	Nonrandomized interventional study	Preschool and school-aged children	Cognitive and visual-motor outcomes	Refractive correction was associated with improvement in selected cognitive and visual-motor measures
Atkinson et al. (2002) [24]	Longitudinal cohort study	5,295 infants with follow-up	Motor and cognitive development	Infant hyperopia predicted poorer visuocognitive and visuomotor performance during follow-up
Alem et al. (2021) [25]	Cross-sectional study	152 Ethiopian schoolchildren	Academic grades	Reduced habitual visual acuity was associated with poorer academic performance
Olatunji et al. (2019) [26]	Cross-sectional study	274 Nigerian schoolchildren	Academic test scores	Uncorrected refractive error was associated with lower academic scores
Latif et al. (2022) [27]	School-based cross-sectional study	High school children in Lahore	Academic performance	Refractive error and correction status were associated with differences in academic performance
Dirani et al. (2010) [28]	Cross-sectional prevalence study	Singaporean children	Refractive error prevalence	The study described the prevalence profile of refractive error and provided an epidemiologic context
Al Bahhawi et al. (2018) [29]	Cross-sectional prevalence study	396 Saudi schoolboys in Jazan	Refractive error prevalence and associated factors	Refractive error was common, supporting the need for school-based detection strategies
van Rijn et al. (2014) [30]	Randomized crossover trial	36 hyperopic children	Reading speed	Full hyperopic correction improved reading speed compared with partial or no correction
Dudovitz et al. (2020) [31]	Quasi-experimental study	Low-income students in the United States	Academic performance	Receipt of corrective lenses was associated with improvement in English language arts performance during follow-up
Nguyen et al. (2025) [32]	Prospective school-based study	647 Vietnamese adolescents	Grade point average and subject scores	Spectacle wear was associated with higher academic performance after adjustment for measured confounders
Wang et al. (2017) [33]	Cluster randomized controlled trial	Rural Chinese schools	Mathematics test scores	Free spectacle provision improved mathematics scores by approximately 0.1 standard deviations compared with control
Ma et al. (2014) [34]	Cluster randomized controlled trial	Rural Chinese schools	Educational outcomes	Free glasses improved educational outcomes modestly, although effects were influenced by adherence
Nie et al. (2020) [35]	Randomized field experiment	Rural Chinese junior high schools	Exam scores, aspirations, and dropout	Free eyeglasses improved exam performance and favorable education-related outcomes
Mayro et al. (2018) [36]	Program evaluation	School-based vision program in the United States	Program implementation and early outcomes	The study described real-world implementation performance and early program outcomes
Bruce et al. (2018) [37]	Cluster randomized controlled trial	UK primary schools	Academic attainment	Free eye examinations and glasses improved spectacle wear but did not clearly improve overall academic attainment
Jiang et al. (2021) [38]	Mendelian randomization study	UK Biobank-based analysis	Educational attainment	The study did not support a clear causal effect of moderate hyperopia on years of education
Halim et al. (2020) [39]	Cross-sectional study	Malaysian schoolchildren	Academic performance	Refractive error was associated with academic performance, although reported effect details were limited
Ma et al. (2021) [40]	Development-effectiveness analysis	Two randomized trials from China	Academic outcomes and delivery models	Educational impact varied by vision care delivery model and context.
Bruce et al. (2016) [41]	Cohort-nested cross-sectional study	11,186 screened UK children; 2,025 included in adjusted analyses	Early literacy	Worse presenting visual acuity at school entry was associated with lower literacy scores after adjustment

Results

Observational Evidence: Early Cognitive, Literacy, and Academic Outcomes

Smaller observational studies supported similar patterns: Shankar et al. [19] reported poorer letter and word recognition and receptive vocabulary among hyperopic kindergarten children, while phonological awareness appeared less affected. In school-aged populations, Williams et al. [20] reported lower standardized attainment among children with hyperopia above a defined threshold, and Rosner and Rosner [21] found lower achievement outcomes among hyperopic children compared with emmetropic peers. Developmental follow-up data also suggested persistence of functional differences, as Atkinson et al. [24] found that hyperopic infants performed worse than controls on visuocognitive and visuomotor measures across early follow-up intervals. Overall, this observational evidence supports an association between uncorrected refractive error and poorer learning-related outcomes, although the strength of inference is limited by confounding, variable exposure definitions, and differences in outcome measurement.

Multiple school-based observational studies also reported associations between refractive error, reduced habitual visual acuity, and academic performance. Alem et al. [25] found that reduced habitual visual acuity was associated with poorer school performance in Ethiopian students, and Olatunji et al. [26] reported that Nigerian pupils with uncorrected refractive error performed worse on academic testing than peers without refractive error. Additional cross-sectional evidence linked refractive error or vision status to school performance in other settings, including Latif et al. [27] and Halim et al. [39]. Prevalence studies provided important context rather than direct evidence of educational effects: Dirani et al. [28] described refractive error prevalence profiles in Singaporean children, and Al Bahhawi et al. [29] reported refractive error prevalence and associated factors in Saudi schoolboys. Bruce et al. [41] linked presenting visual acuity at school entry with literacy performance after adjustment. Together, these cross-sectional and longitudinal findings suggest that visual status may be educationally relevant across settings and over time, but they should be interpreted primarily as associative rather than causal evidence.

Nonrandomized Interventional, Experimental, and Implementation Evidence

Nonrandomized and short-horizon experimental studies provided supportive evidence that visual correction or induced blur can influence near-task performance, but the certainty of this evidence was lower than that of randomized trials. Ntodie et al. [18] found that full hyperopic correction improved accommodative accuracy during sustained near tasks and increased reading speed. Narayanasamy et al. [22] showed that simulated hyperopia during near work reduced reading-related performance, supporting a plausible functional pathway. Roch-Levecq et al. [23] reported improvements in cognitive and visual-motor outcomes after spectacle correction. However, the nonrandomized design of these studies means that practice effects, maturation, task familiarity, and residual confounding may partly explain observed changes. In pragmatic settings, Dudovitz et al. [31] reported improved English language arts performance after receipt of corrective lenses, while Nguyen et al. [32] found that spectacle wear was associated with higher grade point averages and subject scores after adjustment. Mayro et al. [36] provided implementation-focused evidence from a school-based vision program, showing that uptake, logistics, and early program outcomes are important for understanding real-world impact. Ma et al. [40] further showed that educational impact may vary by delivery model and context. Overall, this body of evidence supports plausibility and implementation relevance, but causal certainty remains limited because most studies were not randomized and were vulnerable to selection bias, adherence issues, and confounding.

Randomized Interventional Evidence

Randomized studies provided the strongest evidence for the effect of spectacle correction, although the magnitude and consistency of benefit varied across contexts. van Rijn et al. [30] reported improved reading speed with full hyperopic correction in a randomized crossover trial. Cluster randomized trials in rural China showed that free spectacle provision improved mathematics or educational outcomes, with Wang et al. [33] and Ma et al. [34] reporting modest gains of approximately 0.1 standard deviations in some academic outcomes. Nie et al. [35] also found that free eyeglasses improved exam performance and education-related outcomes, such as aspirations and dropout rates. In contrast, Bruce et al. [37] found improved spectacle wear but no clear improvement in overall academic attainment in a UK cluster trial. Overall, randomized evidence supports a modest potential educational benefit of spectacle provision, but the effect appears implementation-dependent and may be influenced by adherence, follow-up duration, baseline refractive severity, and school context.

Causal Triangulation

To address residual confounding inherent in observational studies, Jiang et al. [38] used Mendelian randomization and did not support a clear causal effect of moderate hyperopia on years of education. This finding cautions against interpreting all observational associations as direct causal effects and reinforces the need to distinguish refractive error as a potential contributor to learning difficulty from broader social, developmental, and educational determinants. Overall, the evidence suggests that uncorrected refractive error is associated with poorer educational outcomes and that spectacle correction can produce modest benefits in some settings. However, the strength of evidence is highest for randomized intervention studies and more limited for cross-sectional and nonrandomized evidence.

Risk of Bias

**Figure 2:**
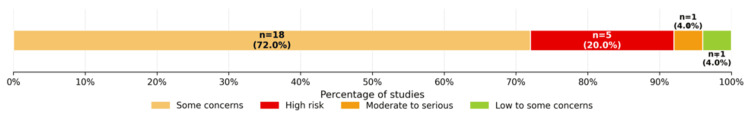
Overall risk-of-bias summary This figure summarizes the overall risk-of-bias judgments across the 25 included studies. Most studies were judged as having some concerns, while five studies were judged to be at high risk of bias. One study was judged as moderate-to-serious risk, and one study was judged as low-to-some concerns. This distribution supports cautious interpretation of the evidence, particularly for cross-sectional, nonrandomized, and program evaluation studies.

Overall, cross-sectional analytical studies were most vulnerable to confounding and selection bias, as refractive status and educational outcomes may co-vary with socioeconomic status, parental education, baseline ability, and access to eye care. Most cross-sectional designs also relied on single-timepoint measurement of both exposure and outcome, limiting causal inference. Across all 25 included studies, five were judged to be at high risk of bias, representing 20.0% of the evidence base. High-risk judgments were assigned to two observational studies and three nonrandomized or program evaluation studies. One study was judged as at moderate-to-serious risk, one as at low risk or with some concerns, and the remaining 18 studies were judged as having some concerns. The most frequent concerns were residual confounding, selection bias, incomplete control for baseline educational differences, limited masking, uncertainty in adherence, and incomplete follow-up reporting (Figure 2).

The risk-of-bias profile of observational studies is summarized in Table 3.

**Table 3:** Risk-of-bias assessment for observational studies (n = 13) Overall judgment categories were low-to-some concerns, some concerns, or high. Tools applied were the Joanna Briggs Institute (JBI) critical appraisal tools for cross-sectional and prevalence studies and the Newcastle-Ottawa Scale (NOS) for cohort studies. Source: [17,19-21,24-29,32,39,41].

Study	Design category and tool	Key bias domains (most relevant)	Overall judgment
Kulp et al. (2016) [17]	Cross-sectional, JBI	Confounding; selection mechanisms; outcome measurement variability	Some concerns
Shankar et al. (2007) [19]	Cross-sectional, JBI	Very small sample; limited confounding control; selection bias	High
Rosner and Rosner (1997) [21]	Cross-sectional, JBI	Confounding; exposure thresholding; selection bias	Some concerns
Alem et al. (2021) [25]	Cross-sectional, JBI	Confounding; socioeconomic and educational factors; outcome grading comparability	Some concerns
Olatunji et al. (2019) [26]	Cross-sectional, JBI	Confounding; school sampling; outcome measurement	Some concerns
Latif et al. (2022) [27]	Cross-sectional, JBI	Confounding by school type and socioeconomic status; unclear temporality for improvement	High
Al Bahhawi et al. (2018) [29]	Cross-sectional, JBI	Confounding; male-only sample; measurement standardization	Some concerns
Halim et al. (2020) [39]	Cross-sectional, JBI	Confounding; outcome reporting completeness	Some concerns
Dirani et al. (2010) [28]	Prevalence, JBI	Sampling representativeness; measurement protocol consistency; response coverage	Low-to-some concerns
Williams et al. (2005) [20]	Cohort, NOS	Confounding; outcome measurement standardization; attrition reporting	Some concerns
Atkinson et al. (2002) [24]	Cohort, NOS	Confounding; loss to follow-up; measurement consistency over time	Some concerns
Nguyen et al. (2025) [32]	Cohort, NOS	Confounding; adherence measurement; residual confounding	Some concerns
Bruce et al. (2016) [41]	Cohort-nested cross-sectional, NOS	Confounding; attrition; outcome standardization across years	Some concerns

Cohort studies strengthened temporality but remained susceptible to residual confounding and, in some reports, incomplete documentation of attrition and follow-up, which may bias educational outcome estimates, as shown in Table 3. Randomized studies reduced baseline confounding, but most were constrained by the limited feasibility of masking, contamination in school settings, and variable adherence to spectacle wear, which may dilute measurable educational effects even when correction is beneficial. Nonrandomized interventional and quasi-experimental studies were generally at higher risk of bias due to nonrandom allocation, selection mechanisms related to educational disadvantage, and time-varying confounding. The Mendelian randomization study reduced conventional confounding but depended on genetic instrument validity, including the absence of pleiotropy and population stratification. The risk-of-bias profile of interventional and causal-inference studies is summarized in Table 4.

**Table 4:** Risk-of-bias assessment for interventional and causal-inference studies (n = 12) Overall judgment categories were some concerns, moderate to serious, or high. Tools applied were RoB 2 for individually randomized and crossover designs, RoB 2 cluster for cluster randomized trials, ROBINS-I for nonrandomized interventional and quasi-experimental designs, and ROB-MR for Mendelian randomization. Source: [18,22,23,30,31,33-38,40].

Study	Design category and tool	Key bias domains (most relevant)	Overall judgment
Ntodie et al. (2021) [18]	Nonrandomized intervention, within-subject pre-post, ROBINS-I	Confounding from learning or fatigue; task order deviations; outcome measurement; selective reporting	Moderate to serious
van Rijn et al. (2014) [30]	Randomized crossover trial, RoB 2	Period or carryover effects; masking not feasible; assessor masking unclear	Some concerns
Nie et al. (2020) [35]	Randomized field experiment, RoB 2	Adherence; masking not feasible; attrition and differential follow-up	Some concerns
Wang et al. (2017) [33]	Cluster randomized trial, RoB 2 cluster	Recruitment bias; deviations and adherence; missing data; unmasked assessment	Some concerns
Ma et al. (2014) [34]	Cluster randomized trial, RoB 2 cluster	Recruitment bias; adherence; contamination; unmasked assessment	Some concerns
Bruce et al. (2018) [37]	Cluster randomized trial, RoB 2 cluster	Adherence; contamination; outcome timing; unmasked assessment	Some concerns
Narayanasamy et al. (2015) [22]	Experimental simulation, ROBINS-I	Selection; performance effects; small sample; task learning	Some concerns
Roch-Levecq et al. (2008) [23]	Nonrandomized interventional, ROBINS-I	Confounding; maturation or practice effects; regression to the mean	High
Dudovitz et al. (2020) [31]	Quasi-experimental, ROBINS-I	Confounding; selection into intervention; missing data over follow-up	High
Mayro et al. (2018) [36]	Program evaluation, ROBINS-I	Selection; confounding; outcome definition and measurement differences	High
Ma et al. (2021) [40]	Secondary analysis of trials, ROBINS-I	Indirectness; model assumptions; selective reporting risk	Some concerns
Jiang et al. (2021) [38]	Mendelian randomization, ROB-MR	Instrument strength; pleiotropy; population stratification; sample overlap	Some concerns

Discussion 

Summary of Key Findings

This systematic review found that uncorrected refractive errors, particularly hyperopia in early childhood, were generally associated with poorer early literacy, attention, visual-motor, and academic outcomes. The most consistent early-childhood signal came from studies of hyperopia, in which reduced near-visual function appeared to coincide with weaker foundational learning skills. Kulp et al. [17] reported poorer early literacy performance among hyperopic preschoolers with measurable near-vision deficits, while Ntodie et al. [18] provided additional evidence that hyperopic children may also show poorer sustained attention and visual-motor integration. These findings support the clinical and educational relevance of early-childhood hyperopia, but they should be interpreted cautiously because most early evidence remains observational and may be influenced by developmental, socioeconomic, and school-level factors.

Strength of Evidence by Study Design

The observational evidence was useful for identifying associations, but it did not establish causality. Shankar et al. [19], Williams et al. [20], and Rosner and Rosner [21] reported poorer emergent literacy or educational attainment among children with hyperopia. These findings are directionally consistent, but the strength of inference is limited by cross-sectional designs, small samples in some studies, variable hyperopia thresholds, and incomplete control for confounding. Mechanistic and developmental studies strengthened biological plausibility. Narayanasamy et al. [22] showed that simulated hyperopic blur can impair reading-related performance, Roch-Levecq et al. [23] reported improved cognitive and visual-motor outcomes after correction, and Atkinson et al. [24] linked early refractive status with later visuocognitive and visuomotor outcomes. However, nonrandomized designs remain vulnerable to maturation effects, practice effects, and regression to the mean.

School-based observational studies from resource-constrained settings supported the public health relevance of uncorrected vision problems. Alem et al. [25] and Olatunji et al. [26] linked reduced habitual visual acuity or uncorrected refractive error with poorer academic performance, while Latif et al. [27] reported performance differences by refractive error and correction status. These studies reflect real school environments, but their findings remain primarily associative. Incomplete adjustment for school type, baseline academic performance, parental education, household income, and access to eye care may partly explain observed differences. Prevalence studies by Dirani et al. [28] and Al Bahhawi et al. [29] did not directly test educational effects, but they show that refractive error is sufficiently common to justify school-based detection and referral pathways.

Interventional evidence provided stronger support for the potential benefit of correction, although effects were generally modest and context-dependent. van Rijn et al. [30] showed improved reading speed with full hyperopic correction in a randomized crossover design. Dudovitz et al. [31] reported improved academic performance after receipt of corrective lenses in a pragmatic setting, and Nguyen et al. [32] found that spectacle wear was associated with higher academic performance after adjustment. These studies suggest that correction may be beneficial, but nonrandom receipt of glasses, adherence measurement, and time-varying school factors limit causal certainty in nonrandomized designs.

The strongest causal evidence came from randomized spectacle provision programs. Wang et al. [33] and Ma et al. [34] reported modest educational gains after free spectacle provision in rural Chinese school settings, while Nie et al. [35] found improvements in academic performance and education-related outcomes. These trials support a potential causal benefit of correcting refractive error, but the magnitude and consistency of benefit appear dependent on implementation quality. Factors such as follow-up systems, compliance, replacement pathways, teacher reinforcement, family support, and social acceptability of spectacle wear may influence whether correction translates into measurable educational gains. Mayro et al. [36] added implementation-focused evidence showing that school vision programs depend on logistics, uptake, and follow-through. Bruce et al. [37] was particularly important because it showed that improved spectacle wear and visual outcomes may not produce detectable short-term academic gains, emphasizing that educational attainment is a distal outcome influenced by many parallel factors.

Mechanisms Linking Refractive Error and Learning

The association between refractive error and learning outcomes is biologically plausible. Moderate hyperopia can reduce near-retinal image quality and increase accommodative demand during reading, writing, and classroom tasks. This may lead to visual discomfort, reduced sustained attention, and lower reading efficiency. These mechanisms are especially relevant in preschool and early primary school years, when children are developing letter recognition, visual-motor coordination, and early reading skills. However, refractive error is unlikely to act in isolation. Its educational impact may be amplified or attenuated by classroom demands, baseline ability, access to correction, spectacle adherence, and the level of support provided by teachers and families.

Residual Confounding, Reverse Causality, and Mendelian Randomization

Residual confounding remains one of the main limitations of this evidence base. Educational outcomes are shaped by socioeconomic status, parental education, nutrition, school quality, home learning support, baseline cognitive ability, and access to health services. These factors may also influence whether a child receives vision screening, obtains spectacles, and wears them consistently. Therefore, observational associations between refractive error and academic performance may partly reflect shared social and educational determinants rather than a direct visual pathway alone.

Reverse causality is particularly relevant for myopia. Academic pressure, near work, and educational exposure may contribute to myopia development, meaning that the direction of association may run partly from education to refractive status rather than solely from refractive status to education. Jiang et al. [38] therefore provide an important cautionary signal. Their Mendelian randomization analysis did not support a clear causal effect of moderate hyperopia on years of education. This finding does not negate the possibility that uncorrected refractive error affects near-task performance or classroom participation, but it does argue against interpreting all observational associations as direct causal effects. It supports a more cautious interpretation in which refractive error is one potentially modifiable factor within a broader educational and developmental system.

Implementation and Policy Implications

The findings have practical implications for school health programs. Screening alone is unlikely to improve educational outcomes unless it is linked to referral completion, affordable or free spectacle provision, adherence support, and follow-up. Program effectiveness may depend as much on delivery quality as on optical correction itself. In low-resource school-based settings, children may be identified but fail to receive glasses, may receive glasses but not wear them, or may require replacement and ongoing reinforcement over time. This is why implementation factors such as teacher engagement, family counseling, stigma reduction, and monitoring of spectacle wear should be considered core components of school vision programs rather than optional additions.

The evidence also suggests that educational outcomes should not be expected to change immediately after correction. Improvements in visual acuity and spectacle wear may occur before measurable academic gains become apparent. Academic performance is influenced by teaching quality, baseline learning gaps, home support, and follow-up duration. Therefore, trials and programs should measure both proximal outcomes, such as spectacle wear and reading speed, and longer-term educational outcomes, such as grades or standardized test performance.

Limitations of the Evidence Base

Several limitations should be considered when interpreting this review. First, studies varied substantially in refractive error definitions, age groups, correction status, outcome measures, and follow-up duration, which prevented meta-analysis. Second, many observational studies were vulnerable to confounding and selection bias, and several did not fully adjust for socioeconomic or educational variables. Third, nonrandomized interventional studies were vulnerable to practice effects, regression to the mean, differential access to correction, and adherence-related bias. Fourth, randomized trials provided stronger evidence but were still affected by limited masking, contamination, variable adherence, and differences in intervention intensity. Fifth, some studies reported associations without detailed effect sizes, limiting comparison across settings. Finally, the evidence base included both ophthalmic and educational outcomes, and these outcomes may operate on different timelines.

Overall Interpretation

Overall, the evidence suggests that uncorrected refractive error is associated with poorer educational outcomes and that spectacle correction can produce modest benefits in some settings. The strongest support comes from randomized spectacle provision trials, while cross-sectional and nonrandomized studies provide useful but lower-certainty associative evidence. Hyperopia in early childhood remains clinically important because of its plausible effects on near-task performance, attention, and early literacy. However, the available evidence should not be interpreted as demonstrating a simple or uniform causal pathway from refractive error to academic underperformance. Future studies should standardize refractive error thresholds and educational outcomes, rigorously measure adherence, adjust for baseline academic and socioeconomic factors, and evaluate implementation strategies that support sustained spectacle wear in real-world school settings.

## Conclusions

Evidence from the included studies suggests a possible association between uncorrected refractive errors in childhood and poorer early literacy or academic performance, with hyperopia showing a relatively consistent relationship with near-task educational domains. However, this association should be interpreted cautiously because of heterogeneity in study designs, refractive error definitions, outcome measures, and risk-of-bias profiles.

Spectacle correction and school-based provision programs may yield modest educational benefits in some settings, but the magnitude and consistency of these benefits appear to depend on adherence, follow-up duration, implementation intensity, and educational context. Future research should prioritize standardized outcome measurement, longer follow-up, rigorous adjustment for confounding, and implementation designs that explicitly address sustained spectacle wear and contextual barriers to uptake.

## Appendices

Supplementary material 1 

**Table 5:** Full database-specific search strategies Search strategies were adapted to the syntax of each database. No language restriction was applied at the search stage. The review included peer-reviewed published studies, while unpublished studies, dissertations, theses, grey literature, and conference abstracts without sufficient data were not included.

Database	Date of final search	Search strategy
PubMed	Jan 31, 2025	("hyperopia"[MeSH Terms] OR hyperopia[Title/Abstract] OR hypermetropia[Title/Abstract] OR "myopia"[MeSH Terms] OR myopia[Title/Abstract] OR "astigmatism"[MeSH Terms] OR astigmatism[Title/Abstract] OR anisometropia[Title/Abstract] OR "refractive errors"[MeSH Terms] OR "refractive error"[Title/Abstract] OR "refractive errors"[Title/Abstract]) AND ("child"[MeSH Terms] OR "adolescent"[MeSH Terms] OR child*[Title/Abstract] OR adolescent*[Title/Abstract] OR student*[Title/Abstract] OR preschool*[Title/Abstract] OR schoolchild*[Title/Abstract] OR pupil*[Title/Abstract]) AND (academic[Title/Abstract] OR education*[Title/Abstract] OR literacy[Title/Abstract] OR reading[Title/Abstract] OR cognitive[Title/Abstract] OR cognition[Title/Abstract] OR "school performance"[Title/Abstract] OR "academic performance"[Title/Abstract] OR "educational attainment"[Title/Abstract]) AND (spectacle*[Title/Abstract] OR glasses[Title/Abstract] OR eyeglasses[Title/Abstract] OR "vision screening"[Title/Abstract] OR correction[Title/Abstract] OR corrected[Title/Abstract] OR uncorrected[Title/Abstract])
Embase	Jan 31, 2025	('hyperopia'/exp OR hyperopia:ti,ab OR hypermetropia:ti,ab OR 'myopia'/exp OR myopia:ti,ab OR 'astigmatism'/exp OR astigmatism:ti,ab OR anisometropia:ti,ab OR 'refractive error'/exp OR 'refractive error':ti,ab OR 'refractive errors':ti,ab) AND ('child'/exp OR 'adolescent'/exp OR child*:ti,ab OR adolescent*:ti,ab OR student*:ti,ab OR preschool*:ti,ab OR schoolchild*:ti,ab OR pupil*:ti,ab) AND (academic:ti,ab OR education*:ti,ab OR literacy:ti,ab OR reading:ti,ab OR cognitive:ti,ab OR cognition:ti,ab OR 'school performance':ti,ab OR 'academic performance':ti,ab OR 'educational attainment':ti,ab) AND (spectacle*:ti,ab OR glasses:ti,ab OR eyeglasses:ti,ab OR 'vision screening':ti,ab OR correction:ti,ab OR corrected:ti,ab OR uncorrected:ti,ab)
Scopus	Jan 31, 2025	TITLE-ABS-KEY((hyperopia OR hypermetropia OR myopia OR astigmatism OR anisometropia OR "refractive error" OR "refractive errors") AND (child* OR adolescent* OR student* OR preschool* OR schoolchild* OR pupil*) AND (academic OR education* OR literacy OR reading OR cognitive OR cognition OR "school performance" OR "academic performance" OR "educational attainment") AND (spectacle* OR glasses OR eyeglasses OR "vision screening" OR correction OR corrected OR uncorrected))

Supplementary material 2

**Table 6:** PRISMA 2020 checklist This table summarizes the PRISMA 2020 reporting checklist for this systematic review. Each item indicates where the corresponding PRISMA requirement is addressed in the manuscript. Items marked as reported were addressed in the main text, tables, figures, or appendices. Items marked as addressed narratively indicate areas where formal quantitative assessment was not performed because of methodological heterogeneity, absence of meta-analysis, or journal format constraints. Declarations items should be completed according to the final journal submission requirements.

PRISMA section	Item number	Checklist item	Location in manuscript	Reporting status
Title	1	Identify the report as a systematic review	Title	Reported
Abstract	2	Provide a summary covering background, objectives, methods, results, limitations, and conclusions	Abstract	Reported in unstructured Cureus review format
Introduction	3	Describe the rationale for the review in the context of existing knowledge	Introduction and Background	Reported
Introduction	4	Provide an explicit statement of the objective or question addressed	End of Introduction and Background	Reported
Methods	5	Specify eligibility criteria for the review, including population, exposure or intervention, comparator, outcomes, and study designs	Eligibility criteria and Table 1	Reported
Methods	6	Specify all information sources searched or consulted	Search strategy paragraph	Reported
Methods	7	Present the full search strategies for all databases and information sources	Supplementary material 1	Reported
Methods	8	Specify the methods used to decide whether each study met inclusion criteria, including the number of reviewers	Data extraction and synthesis	Reported
Methods	9	Specify the methods used to collect data from reports, including the number of reviewers	Data extraction and synthesis	Reported
Methods	10	List and define all outcomes and other variables for which data were sought	Eligibility criteria and Data extraction and synthesis	Reported
Methods	11	Specify the methods used to assess risk of bias in the included studies	Risk-of-bias assessment	Reported
Methods	12	Specify the effect measures sought for each outcome	Data extraction and synthesis	Reported where available
Methods	13	Describe the methods used to synthesize results, including criteria for grouping studies	Data extraction and synthesis	Reported
Methods	14	Describe any methods used to assess risk of bias due to missing results in a synthesis	Risk-of-bias assessment and Discussion limitations	Addressed narratively
Methods	15	Describe any methods used to assess certainty or confidence in the body of evidence	Risk-of-bias assessment and Discussion	Addressed narratively; formal GRADE was not performed
Results	16	Describe the results of the search and selection process, ideally using a flow diagram	Study selection and Figure 1	Reported
Results	17	Cite each included study and present its characteristics	Study characteristics and Table 2	Reported
Results	18	Present assessments of risk of bias for each included study	Risk of bias, Figure 2, Table 3, and Table 4	Reported
Results	19	Present results for each outcome for each study, including effect estimates where available	Main findings and Table 2	Reported narratively
Results	20	Present results of each synthesis conducted	Main findings	Reported narratively
Results	21	Present assessments of risk of bias due to missing results where applicable	Risk of bias and Discussion limitations	Addressed narratively
Results	22	Present assessments of certainty or confidence in the body of evidence	Risk of bias and Discussion	Addressed narratively; formal GRADE was not performed
Discussion	23	Provide a general interpretation of the results in the context of other evidence, including limitations and implications	Discussion	Reported
Other information	24	Provide registration information for the review and state where the protocol can be accessed	Review section, first paragraph	Protocol available from authors upon request; review was not prospectively registered
Other information	25	Describe sources of financial or nonfinancial support and the role of funders	Declarations section	To be completed before final submission
Other information	26	Declare competing interests of review authors	Declarations section	To be completed before final submission
Other information	27	Report which data, code, and other materials are publicly available and where they can be found	Appendix and Results tables	Search strategies are provided in Supplementary material 1; extracted data are summarized in manuscript tables
